# Cause of the Degeneracy of Teeth, Especially among Children

**Published:** 1872-03

**Authors:** S. P. Cutler


AMERICAN JOURNAL

OF

DENTAL SCIENCE.

Vol. V. THIRD SERIES--MARCH, 1872. No. 11.

### ARTICLE I.

### *Cause of the Degeneracy of Teeth, Especially Among Children.*

BY S. P. CUTLER, M.D., D.D.S.

Read before the California State Dental Association.

In attempting to give a satisfactory and rational solution of this question, I find the task a difficult one; a subject embracing both cause and effect, with limited data to base conclusions upon, notwithstanding the facts present themselves duly broad-cast throughout the country, more especially the Southern country, where my labors have been mostly confined.

In the first place, my observations lead me to the fact than the Americans have poorer teeth than any other people on the globe, and the Southern people have poorer teeth that the Northern, as a general rule. The people in large cities and towns have poorer teeth than in the rural districts, and among the more wealthy and refined, still poorer teeth than the poor laboring classes as a rule, excepting cooks and waiters in such families.

Mode of living, habits and climate, all exert modifying influences on human organizations, which are more strikngly manifest in the dental than in any other hard tissues of the body, these organs being more amenable to local influences than any other, at least more tangible, more especially in the young.

Want of cleanliness of the dental structures comes in for a share of our attention, and though only of secondary importance, should not be overlooked.

Let me in the first place investigate the habits and mode of living of the people of our own country, and of the Southern and Northern in contrast, then the climates.

In the first place, what constitutes a well developed and sound constitution in the young of any country? It is proper food and proper physical development or general hygiene.

I will commence with our fashionable home life, and then our fashionable systems of education. In the large towns and cities we find very little natural life, all being artificial that can be made so; night substituted for day, the time that nature needs rest and slumber for the purposes of nutrition and normal repair of wasted tissues; night being the time most suitable for all this. In fashionable life, children are suffered to keep late hours, retire late and get up late in the morning. Here is the first violation of law which has its penalties.

Very little physical exercise is taken, the most important hygiene for bone and muscle development, as well as all other anatomical and physiological perfectibility.

Unless muscular exercise is fully taken in some form or other, the bones cannot be well developed; they will lack that density and resistance essential to physical endurance.

The muscles which are all more or less attached to the bones, confer great power on the bones as *levers*, to bring about the various forms of locomotion. Where there is much powerful muscular exercise during development, unless overdone, there will be a corresponding density and development of muscles, also of bones. The teeth even partake of the same influence of general osseous and muscular development.

Now how do the facts stand? In our fashionable society, in our cities, there are no boys or girls to be seen, they are all Masters and Misses, and young gentlemen and ladies, early in their teens, making and receiving fashionable calls with as much formal state and stiffness as though they were so many machines wound up for the occasions, going through certain prescribed conventional formalities; no juvenile exuberance and hilarity of spirits and flow of youthful impulses. All such would be considered decidedly vulgar and boorish by the young dignitaries of fashionable society.

This was not formerly so much the case, and law was not violated to the same extent as now, and people had better teeth then than now, had better physical forms, better constitutions, and lived longer. In consequence of physical modification in concert with other modifying influences, diseases and their treatment have undergone distinctive modifications.

Now-a-days the mental development is only studied, and that is pushed to the utmost capacity, at the total neglect of physical development. Boys and girls, when they get together at this day do not go out in the yard and romp and play, and scream like so many wild creatures, as they did forty years ago, in this country at least. I cannot speak of the old world from personal knowledge. There is probably not so much difference there as in this country, neither is there the same amount of degeneration in the dental development there as here.

It was a rare thing to see bad teeth forty years ago, and dentists were few and far between, as they were not in demand and no great necessity existed for their services. Now the rule is bad teeth; then bad teeth was an exception. Children then were not sick so much as now, and did not need the services of a doctor as much as now, as the parents had confidence in the *vis medicatrix naturæ* of their children; now more reliance is placed in medicine than formerly, and less confidence in the constitutions of their children to throw off disease, and truly so too.

Let us first examine the little delicate hands and feet of young ladies and gentlemen of fashionable life. The fingers are white, soft, delicate and tender, and may be bent in any direction, as though they had no bones in them, nothing but cartillage, which is the case to a great extent, the bones not being fully developed and hard as nature intended them to be, in order for them to earn their bread by the sweat of their brows, work being decidedly vulgar and not fashionable now-a-days as formerly.

The bones are not well developed for want of proper physical exercise, which not only exerts a direct hygienic influence on bone development, but on every tissue and function of the entire person, even mental.

Without proper physical exercise, digestion is imperfect, and in consequence every function is impaired, the secretions of the mouth becoming, as a general thing, acid in character, which causes early destruction of the imperfectly developed dentures, the teeth decaying frequently before fairly erupted.

Let us examine into the food of children at the present day. Instead of the good, wholesome, substantial food of former times, the most refined flour is used, and bolted meal, all kinds of tubers and fruits must be peeled clean, so as not to leave a trace of the mineral element on them. The dishes must be highly seasoned, and elaborate in numbers, appetizing dishes must be brought into requisition, in order to tempt the incapacitated stomach to take on what it cannot dispose of in a healthy manner. In this way the enfeebled stomachs are overtasked, in consequence, half digested food is conveyed into the bowels, frequently ending in bowel complaints and other disorders; in consequence doctors stuff must be taken to do what nature should do.

This poorly assimilated food, has to build and repair the tissues, which necessarily does the work imperfectly, the hard tissues being the greatest sufferers of all.

An acid condition of the stomach most frequently accompanies this state of things, which attacks and rapidly destroy the new bone and imperfectly developed teeth.

The constant use of sweet cakes and candies among the better classes of children, especially where cleanliness is not observed, is another local cause of early decay, and in many cases of constitutional disturbance, unfavorable to osseous development.

Any cause greatly disturbing healthy nutrition in the young, is markedly felt in the bones and teeth, which, so far as the bones themselves are concerned, may eventually become perfected by change of habits, which would possibly come too late to save the teeth, as they go off so rapidly that time is not given. The teeth themselves may and do undergo systemic changes for the better in many cases, by great care and vigilance.

Late suppers and tea, as so-called, is another evil that ought to be remedied. Food taken too hot, and also too hastily, is another violation of law, which also has its penalties, without any appeal or habeas corpus.

The eternal washing and bathing of little children, which is so extensively practiced by the wealthy, only enfeebles the young constitution; the dirtiest children as a rule, being the healthiest, other things being equal. Healthy children have healthy and clean mouths whether they observe much care or not, the impress on dental development *in utero.*

Let us suppose an expectant mother to have been raised as above mentioned, in the lap of ease and luxury, with a delicate and enfeebled physical constitution, with soft juicy flesh, small and delicate bones, contracted chest, from tight lacing, and want of proper development, from want of proper physical exercise and respiration in consequence.

Here we have in the above case a fetus being developed under unfavorable auspices for constitutional development.

The *enciente* in such cases, being under the doctor's care, every day or two doctors stuff must be taken as something is constantly going wrong with her, her food does not digest, her stomach is acid, in fact her diathesis is of an acid character. It will be remembered that during gestation the tooth germs are formed of the temporary and most of the permanent teeth, commencing at about the sixth week of utero-gestation, and ending somewhere about twelve months after birth. Developmental processes are continued onward until the complete eruption of the teeth, say about twenty years.

All this time dental changes are taking place in regular serial order, and during all this time, local and general influences act more or less powerfully on dental evolution, perfect general organization being essential to perfect dental development.

The confinement within doors of children, and young females especially, and want of sufficient fresh air, and out door exercise in our cities and large towns, where the rooms are not always properly ventilated, is another fruitful cause of badly decayed bones and teeth, which have been referred to already.

The bad and impure air of large towns, where a large amount of coal is consumed, and a corresponding amount of carbonic acid formed and given up to the atmosphere, besides what is furnished by decomposition of filth and respiration, together with other noxious gases, has an important bearing upon the present subject.

The greater the amount of dilution of the atmosphere, the proportion of oxygen is correspondingly lessened, thereby lessening the needed supply to the lungs, blood and other tissues. Whenever there is a deficiency of oxygen furnished for respiration, there necessarily is a deficiency of oxydation taking place in the tissues, and in consequence of nutrition, and when reduced below certain limits, disease sets in, or a tendency to disease is set up, which, if long continued, ends in disease and death. In the latter event the vital resistance is overcome by the inorganic forces to such an extent, that all vital resistance is yielded up, and the organic elements become inorganic again. This normal play of vital, or *vis*

*vitae* and mineral forces, constitutes physiology and health, the opposite pathology.

I will cite an instance that is at this time under my own observation, of a fashionable shoddy aristocratic family, that are boarding in the same house where I am now boarding.

There are four children, all girls, ranging from six to thirteen years respectively. These four children have a room, twenty-two by twenty-four feet square, one English governess and one English servant. These six persons remain in this room all day, only go down to their meals, and at sunset they all take a walk down Canal street a few squares, probably on an average three-quarters of an hour's walk. I never hear the least noise from this room of romping of any kind.

For months there is no change in this system, these children are at their books most of the time; they take lessons in music from their mother, grandmother and governess.

These children are being crowded with mental exercise and almost total neglect of physical. Now in case these children are allowed to eat of sweetmeats and candies, with neglect of oral cleanliness, beyond a doubt bad teeth will follow.

The system of education of the fashionable female boarding schools, is what has been described above, in speaking of this family. This system is wrong in the extreme. The poor laborers on the plantations, and mechanics that have to take constant physical exercise, with plenty of good, plain and substantial food, including the negroes of the South, all have better teeth on an average, than the wealthier and more luxuriant. One striking proof of what I have stated is, that when negro women are brought from the cotton and corn fields, to serve as cooks and waiters about the houses of the wealthy, both on the plantations and in the towns, that these same negroes and their offspring have bad teeth, as a rule, all owing mainly to change of diet and habits generally, their labors being less physical than while in the fields.

Whether the opening of the wilderness, and converting forest lands into cultivated fields, has any direct or indirect agency in the cause under consideration, it is rather difficult to determine. The change of habits and customs, including the diet of the early pioneers, as witnessed in their descendants at the present day, might be regarded as modifying influence, at least on dental and osseous development. The effect this change has produced on climates is well known, but to what extent this change has had in modifying dental development, lacks data to base an opinion upon, though no doubt it has had more or less influence.

The malarial districts, bordering on the father of waters, and its tributaries and other Southern streams are unfavorable to normal dental development.

The modifying influence of high solar heat and miasmata, is very great in the constitutions of children, and to some extent on adult persons.

In the Southern States, with debilitating high temperature, during long summers, the per cent. of oxygen breathed is much less than in more Northern latitudes where the summers are shorter and more interspersed with changes from hot to cool weather, thereby enabling human beings in those regions to breathe more oxygen, and in consequence consume more food, ensuring a higher degree of nutrition, functional activity, and solidity of constitution.

In the South the complexions of the inhabitants are not fresh and vigorous as in the North, owing to the causes already enumerated, and the dental structures must, necessarily feel the same influence to a certain extent.

To lay the axe at the foot of the right tree, we must look to the enciente female of fashionable life for the chief cause of all the bad teeth among very young children, during their deciduous eruptive periods, and during their continuance in the jaws, as these teeth received their morbid impress of imperfect development during utero-gestation. Not only are the deciduous germs all laid down, during comparatively early periods in the stage, but the permanent cor responding in number to the deciduous also. See Tomes' work on the subject of dental development.

We know that delicate females, especially during utero gestation, labor under an acid diathesis, especially manifested in the mouth by rapid progress of decays, if there be any or a tendency to decay, unusual to them at other times. We may from analogy conclude that the entire diathesis is more acid, or abnormally so, and that the same diathesis is also carried to the new offspring.

If such be the case it is but reasonable to infer that there will be an insufficiency of lime furnished to the foetus, or if furnished, will not be the normal tribasic phosphate of lime, but a bibasic, or an acid phosphate, which is in a soluble condition, and subject to be ushered out of these tissues, if even deposited there, as it cannot undergo hardening either in or out of the body.

Now most any acid containing hydrogen will produce this chemical change in the phosphate, and also decompose the carbonate. The acids formed in the body are necessarily organic acids and contain hydrogen, and may produce this effect.

We recognize in pregnant females as a rule, especially in fashionable life, an acid condition of the mouth, which is very destructive to teeth, and in consequence any decayed teeth decay much more rapidly than at any other time

Some men imagine that the teeth of the expectant mother are dissolved away to furnish lime for the foetus. Nothing could be more fallacious than this idea. It has no foundation whatever in science.

Rachetic or rickety children, no doubt in my mind, inherit the acid diathesis from their mothers, which is, I believe, the sole cause of the want of hardening, or ossifying and of the bones, in consequence they remain soft and cartilaginous to a greater or less extent, which may be remedied by use of mineral focil and alcalgis, that is to say, the food containing the laigest amount of phosphates, which has been alluded to already.

The inflammation of the spine, generally accompanying this disease, is in consequence of the pressure of the vertebra on each other in their unnaturally softened condition.

In summing up the question under consideration, it may be given in these words: The penalties attendant on violation of physical laws, mainly the result of modern civilization in this country. Whenever law is violated penalties are sure to follow as a natural sequence. In order to remedy the evil, mankind must obey law, and the ills that flesh is heir to will disappear. Tight lacing is one of the direst evils connected with the subject under consideration and cannot be too severely censured by all professional men.

---

